# Psychosocial wellbeing and risky health behaviors among Syrian adolescent refugees in South Beirut: a study using the HEEADSSS interviewing framework

**DOI:** 10.3389/fpsyg.2023.1019269

**Published:** 2023-05-02

**Authors:** Youssef Rizk, Reem Hoteit, Beatrice Khater, Jihane Naous

**Affiliations:** ^1^Department of Family Medicine, American University of Beirut Medical Center, Beirut, Lebanon; ^2^Lebanese American University Medical Center Rizk Hospital, Beirut, Lebanon; ^3^Faculty of Medicine, Clinical Research Institute, American University of Beirut, Beirut, Lebanon; ^4^Department of Community Health and Family Medicine, College of Medicine, University of Florida, Gainesville, FL, United States

**Keywords:** adolescents, behavioral problems, depression, HEEADSSS interview, health risk behaviors, refugees, asylum seeker, war and demoralization

## Abstract

**Purpose:**

Adolescent refugees are at risk of mental health disorders and underdiagnosed risky behaviors. Limited research exists in the Middle East and North Africa. This study aims to assess psychosocial wellbeing and risk-taking behaviors among adolescent refugees displaced to South Beirut following a standardized framework.

**Methods:**

A cross-sectional study using face-to-face confidential HEEADSSS (Home, Education/Employment, Eating, Activities, Drugs, Sexuality, Safety and Suicide/Depression) interviews was conducted among 52 Syrian adolescent refugees, between the ages of 14 and 21, in a health center in South Beirut.

**Results:**

The mean age of the interviewees was 17.04 ± 1.77 years, with a male predominance 34 (65.4%). Five (9.6%) were married, 38 (73.1%) were not attending school 27 (52.9%) lived in a place with a crowding index ≥3.5 and 21 (40.4%) were working. Risky health concerns or behaviors detected included no activities or exercise 38 (73.1%), eating one to two meals per day 39 (75%) and smoking 22 (42.3%). Eleven (21.2%) have been ever offered drugs and 22 (42.3%) believed they should carry a weapon for protection. Twenty one out of 32 (65.7%) had major depressive disorders and 33 (63.5%) screened positive for behavioral problems. Exposure to home verbal or physical violence, male gender, smoking, and employment were associated with high scoring in behavioral problems. Smoking and ever been touched in an unwanted way were found to be associated with depression.

**Conclusion and practical implications:**

Implementing the HEEADSSS interviewing assessment within medical encounters with refugee adolescents is one efficient way to detect risky health behaviors and mental health problems. Interventions need to be implemented as early as possible in the refugees’ journey to help them cope and gain resilience. Training health care providers to conduct the questionnaire and delivering brief counseling when required is recommended. Establishing a network of referrals to provide multidisciplinary care to adolescents can be helpful. Obtaining a fund to distribute safety helmets for adolescent motorbike drivers can be a way to reduce injuries. More research among adolescent refugees in multiple settings, including teenagers in the host country, is needed to serve this population better.

## Introduction

The current crises in the Middle East and North Africa have resulted in an enormous surge in asylum seekers worldwide (UNHCR, 2019; Palattiyil et al., 2022). Conflict in Syria reached its 12th year in 2022. The migration of Syrians from civil war urges nations to explore sustainable solutions for resettlement (UNHCR, 2020). Concerns over the psychosocial wellbeing of young people who are forced to leave their countries have sparked international calls for studies to direct the creation of support programs for this vulnerable population (Zamani and Zarghami, 2016; Hirani et al., 2018). About half of all refugees globally are under 18 years old (UNHCR, 2019). As of February 2022, around 840,000 Syrian refugees had been registered by the UNHCR as settling in various areas of Lebanon (UNHCR, 2022). However, estimates by the Lebanese government and several local non-governmental organizations suggest that there are 1.5 million Syrian refugees in Lebanon, the majority of whom are children and young adults (WRMCouncil, 2021). In Lebanon, Syrian adolescent refugees were significantly affected by economic pressure during displacement, resulting in a lack of educational opportunities (Cherri et al., 2017). In general, compared to younger and older age groups, adolescents have unique healthcare needs (Patton et al., 2016), and visit doctors less often than any other age group (Nordin et al., 2010). In adolescent refugees and forcibly displaced youth, the situation is furthermore critical, due to the stressors endured and especially when the host country is of low-income and is already struggling to respond to its population needs. Unfortunately, 80 % of all Syrian refugees are located in neighboring countries (Palattiyil et al., 2022).

Risky health behaviors like substance misuse, poor level of activity, early sexual activity, unintentional injuries, exposure to violence, and many others have caused the majority of morbidity and mortality among young people around the world (Mokdad et al., 2016; Klein et al., 2020). Prevention of those risky behaviors (through screening and counseling) is as important as managing their health consequences (Laporte et al., 2017; Searight, 2018). Higher rates of risky behaviors are expected among adolescent refugees, especially since nearly half do not attend school (Palattiyil et al., 2022). A study conducted in Western Australia on adolescent refugees using a structured interview framework reported a high frequency of health risk behaviors requiring intervention (Hirani et al., 2018). Various interventions have been used to tackle current mental health disorders and risk-taking behaviors in refugees while preventing future ones. These are usually used after addressing urgent diagnoses like high risk for suicide or suspicion of abuse. (Mishori et al., 2017) Using face-to-face interviews (Johnson et al., 2021), meaning-based psychotherapy approaches like demoralization and meaning in life (Costanza et al., 2022), cognitive-behavioral therapy (CBT), and narrative exposure therapy (Slobodin and De Jong, 2015) are effective and can be used as early as possible in the displacement. However, most studies were conducted on adults, and few among children and adolescents (Costanza et al., 2022).

Studies showed that exposure to stressful events in infancy and adolescence is associated with impaired post-life physical and mental health effects, including greater involvement in negative health risk behaviors (Felitti et al., 1998; Patton et al., 2016). Behavioral problems and conduct disorders are risk factors for psychiatric disorders and criminal outcomes in adulthood (Schaeffer et al., 2003; De Sanctis et al., 2012). High resettlement stressors like poverty, insecure immigration status and limitations on work and education can tremendously affect the mental health of adolescent refugees and their future productivity (Song and Teichholtz, 2019). Therefore, every adolescent psychosocial interview must include screening for symptoms of depression (Searight, 2018). Mental health disorders were one of the leading causes of disability-adjusted life-years (DALYs) for ages 10–24 for both sexes, whereas for ages 15–19 and 20–24, depressive disorders were the leading cause for females (Mokdad et al., 2016; Blackmore et al., 2020). Depression, anxiety, post-traumatic stress disorders (PTSD) and behavioral problems are prevalent among this population and vary widely among studies (Sapmaz et al., 2017; Kien et al., 2019).

Studies conducted so far in Lebanon on forced displaced refugees tackled psychological disorders and diseases like depression (Naja et al., 2016; Peconga and Høgh, 2020), PTSD (Peconga and Høgh, 2020), anxiety (Peconga and Høgh, 2020), and eating disorders (Aoun et al., 2019). However, a comprehensive assessment of risky health behaviors was not used, behavioral problems were scarcely reported, and few studies included adolescents under 18. We aimed in our study to collect Syrian adolescent refugees’ psychosocial history and identify risk-taking behaviors following an adolescent interviewing framework as per the American Academy of Family physicians (AAFP) and the American Academy of Pediatrics (AAP) guidelines (Ginsburg and Kinsman, 2014; Klein et al., 2020). We also sought to verify in our population of adolescent refugees the factors usually associated with behavioral problems and depression and to find potential new ones.

## Methodology

### Study design

A cross-sectional study using face-to-face interviews was conducted between August and October 2019 in a healthcare center in South Beirut, where forcibly displaced refugees were located. The center is a site where family medicine residents provide care under supervision as part of their training at the American University of Beirut (AUB). Eligible interviewees were Syrian adolescent refugees between the age of 10 and 21 (as per the adolescence period definition), agreeing to be interviewed confidentially and frequenting the center to ensure continuity of care. After reviewing our interview questionnaire, the healthcare center decided to allow us to interview only adolescents aged 14 to 21. All (100) Syrian refugee adolescents between the ages of 14 and 21 who were receiving their primary health care at the designated medical center were intended to be approached by the research team. The snowball sampling technique was planned to be used to recruit additional participants living in the area. The parents, for adolescents under the age of 18, and the participants above the age of 18, were either approached for recruitment by telephone call when confirming their upcoming appointment or on the day of their appointment. An on-site family medicine-trained resident started conducting the interviews initially, then, for the sake of their time, we hired a clinical psychologist to conduct the interviews. Both were independent of the research team. The participants were not compensated for their time and contribution to the study. There was no fund received to complete the study.

### Ethical consideration

The approval of the institutional review board (IRB) at the AUB was obtained prior to commencing the study. An IRB-approved written informed consent was obtained from adolescents 18 years and above. Assent was obtained from adolescents under 18 years after receiving approval from at least one of the parents. In case of illiteracy, the nurse on site (independent from the research team) was present for consenting/assenting parents/adolescents and to sign the documents. Additionally, the interviews were conducted according to the recommendations established by the AAFP and the AAP (Ham and Allen, 2012). Health concerns identified were referred to the medical center team or to the American University of Beirut Medical Center.

### Study instrument

A demographic survey was used to provide information on the characteristics of the participants. The questionnaire following a standardized interviewing framework was conducted in the Arabic language and was used to collect the psychosocial history and identify the risk-taking behaviors of adolescent refugees. The standardized adolescent health interview was based on the traditional HEEADSSS (Home, Education/Employment, Eating, Activities, Drugs, Sexuality, Safety and Suicide/Depression) framework for conducting a comprehensive psychosocial risk assessment of an adolescent, developed by Goldenring and Cohen (1988) and Klein et al. (2020). Framework means that the sequence of domains, or aspects of life, is respected during the interview, however, the specific questions in each are chosen from a set of suggested questions (Table 3 of reference Klein et al., 2020). The HEEADSSS interview is a practical, time-tested, complementary strategy that physicians can use to build on and incorporate the guidelines into their busy office practices. This is an effective way to engage in conversation with teenagers and address many of the challenges faced by this age group (Smith and McGuinness, 2017). After our proposal submission, the latest version added Strengths as well (Klein et al., 2020) to avoid focusing solely on risks. The interview mnemonic became SSHADESS (Strengths, School, Home, Activities, Drugs, Emotions/Eating, Sexuality, Safety). As for drugs excluding tobacco, any kind of use was followed by the CRAFFT questionnaire, a well-known tool to assess drugs in adolescent care, part of the traditional assessment as well (Goldenring and Rosen, 2004). The health center refused to allow us to administer the sexuality domain. After being translated from English, the questionnaire was piloted in Arabic on a group of adolescent refugees. AUB-IRB approved the Arabic version.

For legal purposes, some questions in various domains had to be modified from the initial questionnaire, as advised by the IRB attorney, to avoid involving the authorities. The legislation in Lebanon demands that any findings of an unlicensed firearm, substance use (other than alcohol or tobacco), or suicide attempt must be reported with no respect for the medical encounter confidentiality.

### Measures

#### Sociodemographic and other factors

Age, working hours per day, hours of exercise per week, age of first-time smoking, number of cigarettes/days, number of hubble-bubbles per week, and PHQ9 total score and scoring of behavioral problems were considered as continuous variables. All other variables were considered as categorical.

#### Depression

The Patient Health Questionnaire (PHQ-2) followed by the PHQ-9 if the PHQ-2 score was ≥2, which are usual parts of the HEEADSSS interview, were used to screen for depression (Kroenke et al., 2001). PHQ-9 is a simple 9-item screening instrument used in community settings to detect depression symptoms. Each item is scored for the previous 2 weeks (0: not at all, 1: several days, 2: more than half the days, and 3: nearly every day). The total score ranges from 0 to 27, with higher values implying more severe depression. A score of 0–4 indicates minimal depression; 5–9 indicates mild depression; 0–14 indicates moderate depression; 15–19 indicates severe depression; and a score of 20–27 indicates severe depression. Depression was used as categorical (yes ≥10: major depression; no ≤9: no major depression). A total score of 10 or higher indicated the probability of major depression, with a sensitivity of 80% and specificity of 92% (Manea et al., 2012; Chin et al., 2014).

#### Behavioral problems

Depression and behavioral problems are prevalent among adolescent refugees and have a major impact on post-life physical and mental health effects, including greater involvement in negative health risk behaviors (Felitti et al., 1998; Patton et al., 2016; Sapmaz et al., 2017; Kien et al., 2019). The behavioral problems outcome consisted of eight questions that were added to our HEEADSSS assessment (questions 59–66, please see questionnaire-Appendix). Although the safety domain in the traditional interview contains few questions about behavioral concerns like engaging in fights, we preferred to conduct a more thorough screening, as it is recommended, when a provider has the time to complete it (Goldenring and Rosen, 2004). Questions related to behavioral problems or conduct disorder were obtained from an AAFP article published in 2018 describing how to screen for conduct disorder in an interview: *Conduct Disorder: Recognition and Management* (Goldenring and Rosen, 2004; Lillig, 2018). Any positive response to the eight questions in the section in the behavioral problems screening would prompt the provider to investigate further for a conduct disorder. For the analysis, the screening tool was studied as a continuous variable (0–8).

#### Data analysis

Sociodemographic characteristics have been summarized using descriptive statistics. Continuous variables. Were expressed as means and standard deviations (SD), and categorical variables as frequencies and percentages. The main dependent variables in our study are behavioral problems and depression. Behavioral problems screening score was used as continuous as one person can have one or more behavioral concerns, and depression was used as categorical. We also pursued to verify in our population of adolescent refugees the factors usually associated with behavioral problems (Lillig, 2018) and depression (Kheirallah et al., 2020) cited in the literature. To assess the association between depression and the independent variables, chi-square (χ^2^)/Fisher’s exact tests were used as depression was categorized. To test the association between behavioral problems (calculated as scores) and the independent variables, *t*-test was used to compare means for the variables normally distributed, and Cohen’s d was used to measure the difference between two group means. Statistical analyses were performed using the Statistical Package for Social Sciences (SPSS, version 28) and R language (Version 4.1.2). Statistical significance was set at *p* < 0.05.

## Results

During the study period, 59 adolescent refugees had appointments or visited the clinic and were found eligible to participate in the research study. Of the eligible patients, two refused to participate, and five did not show up on the interview date. Subsequent interviews were arranged and completed with a total of 52 adolescents.

### Sociodemographic

The sociodemographic characteristics of the study group are represented in Table 1. The mean age (range) was 17.1 ± 1.77 (14 to 21) years. There was a predominance of males 34 (65%) compared to females 18 (35%). Most were single 43 (83%), while five (9.6%) were married. Among the married, two were males, three were females and the five were above the age of 18. Two males and two females were engaged, among them, one male and one female were 15 years old.

**Table 1:** Sociodemographic characteristics of 52 Syrian adolescent refugees displaced to South Beirut interviewed following the HEEADSSS framework—2019.

Variables	Mean	SD	n	%
Age	17.04	1.77	–	–
Gender				
Male	–	–	34	65.4
Female	–	–	18	34.6
Marital status	–	–		
Single	–	–	43	82.7
Married	–	–	5	9.6
Engaged	–	–	4	7.7
Currently attending school	–	–		
Yes	–	–	14	26.9
No	–	–	38	73.1

**Figure 1:**
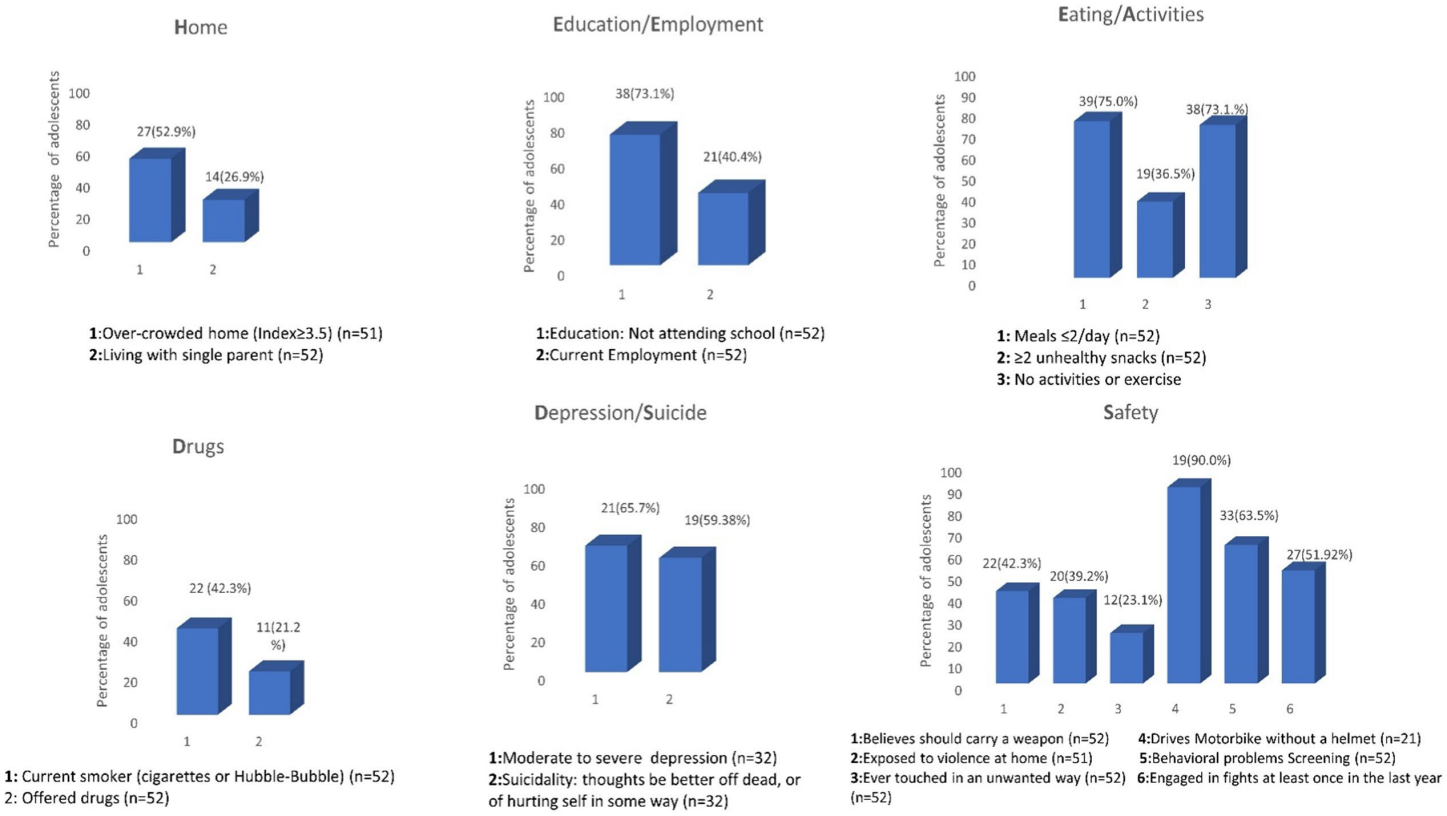
Risky health concerns/behaviors among 52 Syrian adolescent refugees displaced to South Beirut interviewed following the HEEADSSS framework^¥^—2019. ^¥^We reported our findings as possibly similar to the table reported in the AAFP 2020 article reporting risky health behaviors among adolescent high school students in the US (Klein et al., 2020).

**Figure 2:**
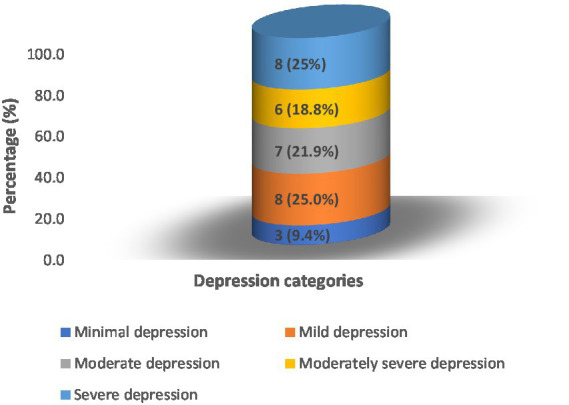
PHQ-9 depression scale among Syrian adolescent refugees displaced to South Beirut (*n* = 32).

The HEEADSSS results are summarized in Figures 1, 2; Tables 1, 2; Supplementary Tables 1A–D.

**Table 2:** Factors found associated to positive screen for depression among 52 Syrian adolescent refugees displaced to South Beirut interviewed following the HEEADSSS framework—2019.

	Depression	
	χ^2^	*p*-value
Gender	0.026	1.000
Exposure to home violence	1.697	0.250
Currently smoker	4.938	0.033**
Currently at school	4.546	0.055
Ever touched in an unwanted way^¥^	6.279	0.017*
Currently employed	0.089	0.781

**Test is significant at the 0.01 level (2-tailed).

*Test is significant at the 0.05 level (2-tailed).

^¥^Fisher exact test.

### Home, education/employment, and eating/activity characteristics

*Home*. More than half of the participants 27 (52.9%) lived in crowded residences (≥3.5). The average number of family members in one household was 7.4 persons (range 5–12). The average number of rooms was 2.1 (range 1–4), and the average total size of the house did not exceed 32 m^2^. Fourteen individuals (26.9%) lived with one of their parents, either their mother or father, while four (7.7%) did not live with any of their parents. *Education/Employment*. Most of the participants were not attending school, 25 males and 13 females (73.1%), none above 18 went to school or college, and 21 (40.4%) were employed (Figure 2). Males tended to start working at an earlier age than females. Sixty-one percent of employed males were under the age of 18, while all working females were above the age of 18. The mean (SD) working hours per day was 8.48 (±2.29) hours. *Eating/Activity* 39 (75%) adolescent refugees had 1–2 meals per day (with an average of 2.2 meals per day among all participants) and a good majority 38 (73.1%) did not exercise or participate in any activity. And 100% of the girls in our sample reported no Activity, hobby, or exercise (Supplementary Table 1A).

### Risky health concerns/behaviors, drugs, suicidality/depression, and safety

*Drugs*. During the previous 12 months of surveying, 4 (7.7%) individuals tried alcohol and 11 (21.2%) were offered drugs other than tobacco and alcohol. The CRAFFT score was administered to these 15 adolescent refugees. Twenty-two (42.3%) of participants were current smokers of cigarettes or hubble-bubble. Further analysis revealed that males smoked cigarettes as well as hubble-bubble, but all females smoked hubble-bubble. The mean age of smoking adolescents was 14.5 ± 2.14 years. The mean number of cigarettes per day was 16.23 ± 6.3 and the number of hubble-bubbles per week per person was 4.25 ± 2.99. Three of the 11 of adolescents (two males and one female) who had been offered any drugs (marijuana, hashish, pills, inhalants, cocaine, or heroin) were under the age of 18 (Supplementary Table 1B). *Suicidality/Depression*. Thirty-two (61.54%) screened positive for depression with a PHQ-2 ≥ 2. Those 32 underwent the PHQ-9 questionnaire. Of the 32 participants, 21 (65.7%) had major depression (score ≥ 10). Fourteen out of 32 (43.75%) scored in the categories of moderately severe and severe depression (score ≥ 15). Of the 32 refugee adolescents screened positive for depression, 19 (59.38%) reported suicidal thoughts. The descriptive statistics of depression are shown in Supplementary Table 1.

*Safety*. Twenty-two adolescent refugees (42.3%) believed they should carry a weapon (knife or gun) to protect themselves. Twelve (23.1%) reported ever being touched in an unwanted way, 20 (39.2%) were ever exposed to verbal or physical violence at home, and 27(51.92%) had engaged in fights in school or the neighborhood at least once in the last year. Twenty-one (40.38%) drove a motorcycle, of whom 19/21 (90.47%) did not wear a safety helmet. Screening for behavioral problems (at least one positive response to the 8 questions administered) was found positive in 33 (63.5%) adolescent refugees (Supplementary Table 1D). The descriptive statistics of the behavioral problems are shown in Supplementary Table 1. Any positive screening in the depression or behavioral problems domains was referred to the mental health providers or physicians in the center or in AUB if needed.

### Factors associated with depression and behavioral problems

Smoking status (*χ*^2^ = 4.938, *p* = 0.033) and ever been touched in an unwanted way (*χ*^2^ = 6.279, *p* = 0.017) were found to be associated with a positive depression screening. Male gender (*t* = 2.909, *p* = 0.005), history of exposure to home violence (*t* = −2.734, *p* = 0.009), smoking status (*t* = −2.112, *p* = 0.040) and current employment (*t* = −1.889, *p* = 0.032) were found associated to a positive screen for behavioral problems in our adolescent refugee population (Tables 3, 4).

**Table 3:** Factors found associated to positive screen for behavioral problems among 52 Syrian adolescent refugees displaced to South Beirut interviewed following the HEEADSSS framework—2019.

	Behavioral problems	
	Independent *t*-test Cohen’s *d*	*p*-value
Gender	2.909 *0.848*	0.005**
Exposure to home violence	−2.734 −*0.784*	0.009**
Currently smoker	−2.112 −*0.593*	0.040*
Currently at school	−0.373 −*0.116*	0.711
Ever touched in an unwanted way	−0.439 −*0.145*	0.662
Currently employed	−1.889 −*0.534*	0.032*

**Test is significant at the 0.01 level (2-tailed).

*Test is significant at the 0.05 level (2-tailed). Italic represents the Cohen’s *d* value.

**Table 4:** Descriptive statistics of the behavioral problems outcome among 52 Syrian adolescent refugees displaced to South Beirut.

	Positive answer* n (%)
1. Have you skipped school in the last 12 months?	7 (13.5)
2. Have you been suspended or expelled from school?	4 (7.7)
3. Have you ever gotten into any physical fights at school?	8 (15.4)
4. Have you gotten into physical fights in your neighborhood, or other places?	25 (48)
5. Have you gotten in trouble with the police?	14 (26.9)
6. Have you been in situations where you destroyed property?	11 (21.1)
7. Have there been times when you stayed out very late without permission?	13 (25)
8. Have there been times when you have run away from home?	7 (13.5)

*An interviewee might answer yes to multiple questions.

## Discussion

Structured interviews, conducted confidentially to assess psychosocial wellbeing and risky health behaviors among adolescent Syrian refugees, helps spot issues and disorders in a center serving refugees in Beirut. Early marriage and employment, low school enrollment, tobacco use, drug access, sexual intimidation, and positive screening for depression and behavioral problems, besides other health concerns, were detected with high frequencies.

Our study’s high rates of health risk behaviors and depression emphasize the obstacles and challenges that the Syrian adolescent population faces in a low-to-middle income host country. We assessed our findings by comparing them to existing literature, examining associated factors, and speculating on what more may be learned about this vulnerable group. Moreover, we provided a series of recommendations for the center to consider because these rates can be used to improve health outcomes.

### Health concerns found prevalent in multiple domains of the HEEADSSS assessment (sexuality omitted)

Comparing our data to the national 2017 US data (Kann et al., 2018) and to similar studies among refugees in high and low-income host countries revealed that our population is at high risk in multiple domains.

Our study showed that 27(52.9%) of the adolescents lived in *Homes* or households with a crowding index above 3.5. The American crowding index defines severe crowding as having more than 1.5 persons per room (WHO, 2018). Crowded homes in studies among Palestinian refugees have been found to be associated with a lack of privacy, the spread of disease, and an increase in home accidents (Al-Khatib et al., 2005; Habib et al., 2006). As for *Eating*, our data is similar to the 2015 UNHCR report (UNHCR, 2015), which found an average of 2.2 meals per day. A healthcare provider doing the screening and counseling in these domains might perceive crowded homes and food type and availability to be non-modifiable factors. However, counseling in early life about reducing the consumption of sodas and unhealthy snacks is doable and is likely to change adolescents’ snack choices. Moreover, communicating housing and food issues with objective and accurate data to authorities and non-governmental organizations is more likely to generate adequate help.

Regarding *Education and employment*. The high rate of dropping out of school 38 (73.1%) in our studied population, is a concerning factor because of the possible link to adverse long-term outcomes (DeJong et al., 2017). A report by the Overseas Development Institute (ODI) on Syrian refugees in Lebanon found that the proportion of children not attending school increases considerably with age, with a reported 92.26% of children 15–18 years old out of school in 2014 (Watkins and Zyck, 2014). The reported barriers in the literature affecting education for refugees are foreign language needs (schools in Lebanon teach French or English with Arabic), interrupted schooling, bullying by the host country’s peers, and academic difficulties (DeJong et al., 2017; Hirani et al., 2018). These were not collected variables in our study. However, child marriage and the female gender were reported in the literature as contributive factors for school interruption (DeJong et al., 2017). Our study found that 100% of the girls in our sample reported no *Activity*, hobby, or exercise, implying some over-protection of girls; however school drop, and marriage were almost similar in both genders of our sample. The small sample size in our study most likely affected the finding of concordant results with the literature. As for employment, 21 (40.4%) of our sample of adolescent refugees were found working an average of 8.5 h per day. Habib et al., reported in a study done among Syrian refugee child workers in the Bekaa valley of Lebanon, an average of 6.7 h per day (Habib et al., 2021). We want to emphasize that our results are alarming, and would like to remind that child labor is illegal, and some tasks and exposures are reported to be harmful to a child’s health at such a young age, with a risk of work-related injuries (Habib et al., 2020, 2021). Further questions need to be directed to families and schools surrounding the center to investigate the dropout from school and the high employability.

Concerning *Drugs*, nearly 22(42.3%) of the adolescent refugees smoked, and almost 11(21.2%) had been ever offered drugs. The reported tobacco use rates in a study with similar background characteristics but a larger sample size among adolescent Palestine refugee and non-refugee groups were 26.7% vs. 24.0% in Jordan, 39.4% vs. 38.5% in Lebanon, and 39.5% vs. 38.4% the West Bank (Jawad et al., 2016). Low alcohol consumption was reported; however, it could be under-reported due to religious stigma, as other studies have described (Baron-Epel et al., 2015). Cigarette and drug misuse should be discussed in private at every visit because counseling and dealing with peer pressure are crucial in preventing future problems.

About *Safety*, only one out of every 10 refugees use a helmet while riding a motorbike and 22(42.3%) believed they need a weapon (knife or gun) to protect themselves. It is well known that the morbidity and mortality from an accident are not negligible and unintentional injuries are prevalent among youth (Goldenring and Rosen, 2004; Searight, 2018; Klein et al., 2020). Awareness campaigns should focus on preventing adolescent motorcycle use without a helmet. Funding might be needed if buying a helmet is outside the reach of the adolescents or their families. The dangers of carrying a weapon should be thoroughly explained as well.

One modifiable *home-*related and *safety*-related risk factor identified in our study would be exposure to violence, reported in almost 40% of homes. However, we did not investigate if these incidents were war-related or not. In a study conducted in Turkey in 2018 about forcibly displaced Syrian children and adolescents, 25.6% personally experienced cruelty during the war (Gormez et al., 2018). Raising awareness about avoiding verbal/physical violence at home and, if possible, preventing children and adolescents from experiencing violent incidents has been shown to reduce morbidity in current and older age groups (Kingston et al., 2016). While we did not go through the details of the exposure to violence before the settlement in Beirut, we recommend concrete efforts to stop violent experiences among this vulnerable population.

### Behavioral problems screening among adolescent refugees

A high score for behavioral problems 33(63.5%) was found in our Syrian adolescent refugees when a set of questions was utilized to screen for the presence of the disorder. We found violence at home and the male gender as the main associated factors. Current cigarette use and current employment were also found to be significantly associated with a positive screen for a behavioral problem. These findings echo the literature where male sex and exposure to physical or sexual abuse or domestic violence were cited as risk factors for behavioral problems, among many others (Lillig, 2018). A study conducted by Çeri and Nasiroğlu (2018) visiting behavioral problems and other mental health disorders among Syrian refugee children in Turkey, reported a positive screening for conduct problems in 21/77(27.3%) and an association to previous traumatic experience. However, it is important to note that, apart from war-related violence, exposure to violence or abuse and substance use are factors for behavioral problems not unique to the Syrian adolescent population (Lillig, 2018). These factors are shared by any adolescent living in these circumstances. Therefore, the frequency identified should be compared to their Lebanese and Palestinian adolescent peers, and the effect of witnessing war-related violence is a factor to be studied solo. Furthermore, because our tool is a screening rather than a diagnostic tool, adolescents who screened positive for behavioral problems should be reassessed by a mental health practitioner and receive appropriate counseling and assistance. We recommend constant screening of behavioral problems among adolescents and specifically vulnerable ones like refugees, as behavioral problems and conduct disorders are predictors of poor psychiatric and criminal outcomes in adulthood (Schaeffer et al., 2003; De Sanctis et al., 2012).

### Depression among adolescents refugees

According to a recent systematic review, the total depression prevalence among refugee children and adolescents is estimated to be 14% (Blackmore et al., 2020). Also, a study conducted in Turkey in 2017, using a psychiatric assessment through interviewing, reported a psychiatric disorder found in almost half of the children and adolescent refugees and a depressive disorder among 13.4% (Sapmaz et al., 2017). Our data demonstrated a high prevalence of depression, 21/32 (65.6%) scored above 10 on the PHQ-9. This could be explained by the fact that the living conditions in a developing country like Lebanon, are difficult as resettlement stressors and school interruptions are high. Lebanon is an unstable country with frequent traumatizing events that trigger mental health disorders in the local and refugee populations. For example, the Beirut Port semi-nuclear explosion of August 2020 was a major traumatic event that increased the need for mental health care in the country. Additionally, witnessing parents and caregivers enduring the displacement and going through adaptation problems make adolescent refugees subject to depression and other mental health problems (Paoletti et al., 2013).

Moreover, we are concerned about the suicidal ideations rate of 19/32 (59.38%). None of the studies cited in our discussion reported any percentage in an adolescent refugee population. However, a recent study among Syrian adult refugees in Lebanon reported 40% of suicidal thoughts (Naal et al., 2021). We fear as well these adolescent refugees are going through suicidal ideations linked to a demoralization and hopelessness process rather than depressive symptomatology since demoralization has been found to be an independent risk factor for suicide regardless of the presence of clinical depression (Clarke and Kissane, 2002; Costanza et al., 2022). Immediate action regarding universal screenings and appropriate referrals is needed to protect these adolescents and maintain their mental health. Finally, our findings revealed that smoking cigarettes and ever been touched in an unwanted way are associated with higher levels of depression. Contemporary research among adolescents and youth refugees in Jordan yielded comparable results regarding tobacco and its association with depression and prior trauma (Kheirallah et al., 2020).

## Limitations

The key drawback of this study is that the sample size might be perceived as low and limiting the study’s power. Due to ethical reasons, the study had to take place in a setting with an appropriate referral and support system if any new health risk was identified, therefore, we could not approach any adolescent refugee in the area. We could not reach adolescents in their homes, thus limiting the sample to those who attend the center. A working adolescent or a girl raised in a conservative milieu might not be accessible. As we were aiming for a convenience sample, we expected to reach more adolescents frequenting the center (100 registered in the center) and more living in the camp using the snow-balling technique. Unfortunately, the study was disrupted by the frequent demonstrations and road closures imposed by the “October 19 revolution” in Lebanon, which lasted many months. It was followed by the formal declaration of the COVID-19 pandemic and lock-down in March 2020. IRB prohibited any face-to-face interviewing throughout the pandemic, and the center could no longer accommodate our presence as it had to deal with the COVID-19 cases.

Despite efforts to ensure that respondents felt comfortable answering sensitive questions and that confidentiality will be preserved, under-reporting of risk behaviors in this conservative setting might be a potential limitation of this study. In addition, several aspects of the data relied on participants recalling past events, which may result in recall bias. However, using the questionnaire in Arabic, which is the native language of the refugees, might have ensured comfort during the encounter as an interpreter did not need to be present in the room. This increased the adolescent’s perception of confidentiality and reduced any information that could be lost in interpretation.

Other limitations were imposed by the setting where the interviews took place and by the legal system in Lebanon as explained earlier in the methods section. Since we dealt with a vulnerable population, the center’s medical and mental health teams strongly recommended excluding younger adolescents (ages 11–13). Moreover, the sexual domain questions were excluded from the survey, as requested by the center, as the parents would not accept these questions as “culturally” appropriate.

## Strengths

To the best of our knowledge, this is the first study in Lebanon and the Middle East that aims to assess adolescent refugees’ psychosocial condition and behavior, including those under 18, using the HEEADSSS adolescent health framework as per the AAFP and the AAP guidelines (Sapmaz et al., 2017; Klein et al., 2020). This study demonstrates the importance of following current guidelines for interviewing adolescents using structured questionnaires. Some health concerns will go unnoticed if the physician does not ask targeted, well-structured questions. Moreover, behavioral problems or conduct disorder screening was fully included in our framework, as we aimed to visit this under-screened domain.

Despite circumstances disrupting the data-gathering process, as explained above, our sample is reliable due to the rigorous methodology we utilized and the comprehensive data we collected. Moreover, most families had no issues interviewing the adolescent alone, and the HEEADSSS questionnaire demonstrated efficiency in this vulnerable population by identifying many health concerns.

## Practical implications

We encourage the routine use of the HEEADSSS assessment in a confidential setting with all refugee adolescents rather than only, when necessary, to detect health risk behaviors and mental health disorders. Interventions need to be implemented as early as possible in the refugees’ journey to help them cope and gain resilience. Training health care providers in the center or any needed similar setting to conduct the questionnaire and delivering brief counseling when required is recommended. Establishing a network of referrals to provide multidisciplinary care to the adolescents in whom risk behaviors and disorders were identified can be helpful and useful. Obtaining a fund to distribute safety helmets for motorbike drivers can be a way to reduce injuries related to motor vehicle accidents. It is necessary to intensify research on adolescents and young adults, to mobilize the medical body, the governmental and non-governmental funds, and social affairs toward specific risk-taking behaviors that may impact adult health outcomes.

## Conclusion

Using standardized questionnaires to assess adolescent Syrian refugees’ health behaviors and psychosocial wellbeing helps detect numerous risk behaviors. It allows healthcare practitioners to better serve the adolescent population of refugees and their peers. It will also assist them in better understanding the needs and behaviors of this population so that they can intervene accordingly. More research with a larger population is needed to draw more precise conclusions and compare with the health risk behaviors and mental health problems of Lebanese adolescents living in a similar neighborhood. In addition, research comparing the behaviors of adolescent refugees displaced to an urban area and adolescent refugees displaced to a rural area is needed.

## Data availability statement

The raw data supporting the conclusions of this article will be made available by the authors, without undue reservation.

## Ethics statement

The approval of the institutional review board (IRB) at the American University of Beirut was obtained prior to commencing the study. Written informed consent to participate in this study was provided by the participants’ legal guardian/next of kin.

## Author contributions

YR and JN designed the study and wrote the proposal. JN and RH analyzed the data. RH prepared the tables and figures. YR, JN, and RH wrote the main manuscript text. BK revised the final draft. All authors contributed to the article and approved the submitted version.

## Acknowledgments

We thank the participants and their families for their time and welcomeness. We also thank Dr. Dany Daham for his coordinating role between the university and the medical center, and Dr. Andrea Mladenovic for her assistance in coordinating with the IRB office and organizing the initial data collection.

## Conflict of interest

The authors declare that the research was conducted in the absence of any commercial or financial relationships that could be construed as a potential conflict of interest.

## Publisher’s note

All claims expressed in this article are solely those of the authors and do not necessarily represent those of their affiliated organizations, or those of the publisher, the editors and the reviewers. Any product that may be evaluated in this article, or claim that may be made by its manufacturer, is not guaranteed or endorsed by the publisher.
