# Supplementary material for: Psychosocial wellbeing and risky health behaviors among Syrian adolescent refugees in South Beirut: a study using the HEEADSSS interviewing framework
Source: Front Psychol. 2023 May 2;14:1019269. doi: 10.3389/fpsyg.2023.1019269 (PMC10187139; doi:10.3389/fpsyg.2023.1019269)
Supplement: Supplementary file 1 [file Table_1.docx]

**Supplementary tables**

HEEADSSS assessment Excluding sexuality, including Behavioral problems screening under safety

**Table S1-A.** **H**ome-**E**mployment-**E**ating Habits-**A**ctivities(exercise)

| Variables | **Mean** | **SD** | **n** | **%** |
| --- | --- | --- | --- | --- |
| **Home** |  | | | |
| Lives with mother only |  |  | 12 | 23.1 |
| Lives with father only |  |  | 2 | 3.8 |
| Lives with both parents |  |  | 34 | 65.4 |
| Lives with other family member |  |  | 4 | 7.7 |
| **Crowding index (co-resident/room)** |  | | | |
| ≤2.5 |  |  | 6 | 11.8 |
| 2.51-3.49 |  |  | 18 | 35.3 |
| ≥3.5 |  |  | 27 | 52.9 |
| **Employed** |  | | | |
| Yes |  |  | 21 | 40.4 |
| No |  |  | 31 | 59.6 |
| Working hours/day | 8.48 | 2.29 |  |  |
| **Eating Habits** |  | | | |
| **Meals** |  | | | |
| 1/day |  |  | 10 | 19.2 |
| 2/day |  |  | 29 | 55.8 |
| 3/day |  |  | 9 | 17.3 |
| 4/day |  |  | 4 | 7.7 |
| **Activities/Exercise** |  |  |  |  |
| Yes |  |  | 14 | 26.9 |
| No |  |  | 38 | 73.1 |
| Hours of exercise | 2.29 | 1.07 |  |  |

**Table S1-B**. Tobacco and **D**rugs

| Variables | **Mean** | **SD** | **n** | **%** |
| --- | --- | --- | --- | --- |
| **Ever smoked Cigarettes** |  | | | |
| Yes |  |  | 25 | 48.1 |
| No |  |  | 27 | 51.9 |
| **Age of first-time smoking** | 14.5 | 2.14 |  |  |
| **Currently Smoking Cigarettes or hubble-bubble** |  | | | |
| Yes |  |  | 22 | 42.3 |
| No |  |  | 30 | 57.7 |
| **Number of cigarettes per day** | 16.23 | 6.30 |  |  |
| **Number of hubble-bubbles per week** | 4.25 | 2.99 |  |  |
| Family members smokers |  |  | 44 | 84.6 |
| Friends-smokers |  |  | 42 | 80.8 |
| Thinking about quitting |  | | | |
| Yes |  |  | 42 | 80.8 |
| No |  |  | 10 | 19.2 |
| **Drugs** |  | | | |
| Ever tried Alcohol |  | | | |
| Yes |  |  | 4 | 7.7 |
| No |  |  | 48 | 92.3 |
| Ever offered drugs other than tobacco and alcohol |  | | | |
| Yes |  |  | 11 | 21.2 |
| No |  |  | 41 | 78.8 |
| Ever driven car when high or ridden with someone high |  | | | |
| Yes |  |  | 2 | 3.8 |
| No |  |  | 50 | 96.2 |
| CRAFFT score administered |  |  | 15/52 |  |
| CRAFFT score ≥ 2 |  |  | 12/15 |  |
| Screening drugs misuse positive |  |  | 12/52 | 23.0 |

**Table S1-C. D**epression/**S**uicidality

| Variables | **Mean** | **SD** | **n** | **%** |
| --- | --- | --- | --- | --- |
| PHQ2≥2 |  |  | 32/52 | 61.54 |
| PHQ9 (N=32) | 13.31 | 6.36 |  |  |
| PHQ9<5 Minimal depression |  |  | 3 | 9.37 |
| 5≤PHQ9<10 Mild depression |  |  | 8 | 25.0 |
| 10≤PHQ9<15 Moderate |  |  | 7 | 21.88 |
| 15≤PHQ9<20 Moderately severe |  |  | 6 | 18.75 |
| PHQ≥20 Severe |  |  | 8 | 25.00 |
| Thoughts better be dead (N=32) |  |  | 19 | 59.38 |

**Table S1-D. S**afety/Behavioral problems screening

| Variables | **Mean** | **SD** | **n** | **%** |
| --- | --- | --- | --- | --- |
| **Exposed to violence at home (verbal-physical)** |  | | | |
| Yes |  |  | 20 | 39.2 |
| No |  |  | 31 | 60.8 |
| **Ever touched in an unwanted way** |  | | | |
| Yes |  |  | 12 | 23.1 |
| No |  |  | 39 | 75 |
| Missing |  |  | 1 | 1.9 |
| **Weapons at home** |  |  |  |  |
| Yes |  |  | 2 | 3.8 |
| No |  |  | 50 | 96.2 |
| **Believes should carry a weapon** |  |  |  |  |
| Yes |  |  | 22 | 42.3 |
| No |  |  | 30 | 57.7 |
| **Wears Helmet on motorbike (N=21)** |  |  |  |  |
| Yes |  |  | 2 | 9.53 |
| No |  |  | 19 | 90.47 |
| **Behavioral problems Screening** | 1.71  (Min0-Max5) | 1.70 |  |  |
| Positive Screen for behavioral problems (at least one positive response out the eight) |  |  |  |  |
| Yes |  |  | 33 | 63.5 |
| No |  |  | 19 | 36.5 |
| One positive answer |  |  | 8/33 | 24.2 |
| Two positive answers |  |  | 8/33 | 24.2 |
| Three positive answers |  |  | 7/33 | 21.2 |
| Four positive answers |  |  | 6/33 | 18.2 |
| Five positive answers |  |  | 4/33 | 12.2 |
