# Supplementary material for: Psychosocial wellbeing and risky health behaviors among Syrian adolescent refugees in South Beirut: a study using the HEEADSSS interviewing framework
Source: Front Psychol. 2023 May 2;14:1019269. doi: 10.3389/fpsyg.2023.1019269 (PMC10187139; doi:10.3389/fpsyg.2023.1019269)
Supplement: Supplementary file 2 [file Data_Sheet_1.docx]

Appendix

**Adolescent Care Assessment**

**HEEADSSS interview questionnaire**

**“The things we talk about here are private, between you and me, and will not be disclosed at any time unless you decide so. The only times I need to discuss it with anyone else is if you tell me that you are at risk of hurting yourself, hurting others or being hurt by others, at this time your approval is still a must.”**

**General Information**

1. **Age**
2. **Sex □** Male **□** Female **□** other
3. **Marital status □** Single **□** Married  **□** Engaged **□** Divorced **□** Widowed

**Home**

4. Who do you currently live with?

☐Mother ☐ Father ☐both parents ☐Grand Parents ☐Step-mother/father ☐Alone ☐other Family member or other

5. How many individuals are you in total at home? _____

6. How many rooms are there at home?

☐1 ☐2 ☐3 ☐4

7. Is there any violence (verbal or physical) at home? ☐Yes ☐No

**Education/Employment**

8**.** Are you currently in school? ☐ Yes ☐ No, In what grade?

9. Are you currently employed? ☐ Yes ☐ No

If yes,

where?

What ?

10. How much time do you work a day (in hours)?

**Eating**

11. How do you look at yourself? ☐Underweight ☐Overweight ☐Obese ☐Of Normal weight

12. How many meals do you have usually per day? ☐1 ☐2 ☐3 ☐4

13. What about snacks? ☐1 ☐2 ☐3 ☐4

14. How much water do you drink a day? ☐500 ml ☐1 L ☐1.5 L ☐ 2 L ☐more than2 L

15. Where do you get your drinking water from? ☐Tap water ☐Bottled water ☐unknown source

16. How many Soft drinks do you drink per day? ☐1 ☐2 ☐3 ☐4

17. Unhealthy snacks (chips, chocolate, biscuits)? ☐1 ☐2 ☐3 ☐4

**Activities**

18. Do you participate regularly in any sports or other activities? ☐Yes ☐No

19. If yes, How many hours do you exercise per week? (Number)

**Drugs**

20. Have you ever smoked cigarettes? ☐ No ☐ Yes

21. Do you currently smoke? ☐ No ☐ Yes ☐ Occasionally

22. If yes, at what age did you start smoking?.........................

23. How many cigarettes do you have per day? _ _

24. How many narguile/shisha do you have per week? _ _

25. Are you thinking about quitting smoking? ☐ No ☐ Yes

26. Do any of your friends use Tobacco? ☐Yes ☐No

27. Do any of your friends use Alcohol? ☐Yes ☐No

28. Do any of your friends use other drugs? ☐Yes ☐No

29. Does anyone in your family use Tobacco? ☐Yes ☐No

30. Does anyone in your family use Alcohol? ☐Yes ☐No

31. Does anyone in your family use other drugs? ☐Yes ☐No

32. What about you? During the PAST 12 MONTHS, did you:

Drink any alcohol (more than a few sips)?

(Do not count sips of alcohol taken during family or religious events.) ☐Yes ☐No

33. If yes how many drinks do you have per week on average? --------

34. Have you ever been offered any type of drugs other than alcohol or tobacco?

☐Yes ☐No

If Patient answers “Yes” to 32 or 34 questions, please administer CRAFFT questionnaire, if all are No Answer administer only question 35

35. Have you ever ridden in a **CAR** driven by someone (including yourself) who was “high” or had been using alcohol or drugs?

☐Yes ☐No

36. Do you ever use alcohol to **RELAX**, feel better about yourself, or fit in?

☐Yes ☐ No

37. Do you ever use alcohol while you are by yourself, or **ALONE**?

☐Yes ☐ No

38. Do you ever **FORGET** things you did while using alcohol?

☐Yes ☐ No

39. Do(es) your **FAMILY or FRIENDS** ever tell you that you should cut down on your drinking?

☐Yes ☐ No

40. Have you ever gotten into **TROUBLE** while you were using alcohol?

☐Yes ☐ No

41. CRAFFT score

*Scoring and Interpretation:*

*Part A: If “yes” to any questions in Part A, ask all 6 CRAFFT questions. If “no” ask CAR question then stop.*

*Part B: Score 1 point for each “YES” answer. CRAFFT Score Degree of problem related to alcohol*

*0-1 No problems reported Suggested Action None at this time.*

*2+ Potential of a significant problem. Suggested Action Assessment required.*

**Sexuality (Omitted by the center, not addressed)**

Have you ever been physically intimate? No Kissing Touching Sexually Active

**If sexually active, ask next questions**

Do you have sex with  Males  Females  both?

In the past 12 months, how many partners have you had sex with? ......................................................

What do you do to protect yourself from HIV and Sexually Transmitted infections? .............................

Have you ever had an STD?  No Yes ………………………………………………………

What contraception methods are you using?

☐None ☐ Calendar Method ☐Withdrawal ☐Condoms ☐OCPs

☐IUD ☐Implant ☐Injectable ☐Patch ☐Ring

**Suicide/depression-PHQ2and 9(if PHQ=>2)**

Over the past two weeks, how often have you been bothered by the following problems?

42. Little interest or pleasure in doing things

0. Not at all 1. Several days 2. More than one-half of the days 3.Nearly every day

43. Feeling down, depressed, irritable, or hopeless

☐0. Not at all ☐1. Several days ☐2. More than one-half of the days ☐3.Nearly every day

(if score is ≥ 2 complete PHQ9 questionnaire)

44. Trouble falling or staying asleep, or sleeping too much

☐0. Not at all ☐1. Several days ☐2. More than one-half of the days ☐3.Nearly every day

45. Feeling tired or having little energy

☐0. Not at all ☐1. Several days ☐2. More than one-half of the days ☐3.Nearly every day

46. Poor appetite, weight loss, or overeating

☐0. Not at all ☐1. Several days ☐2. More than one-half of the days ☐3.Nearly every day

47. Feeling bad about yourself, that you are a failure, or have let yourself or your family down

☐0. Not at all ☐1. Several days ☐2. More than one-half of the days ☐3.Nearly every day

48. Trouble concentrating on things such as schoolwork, reading, or watching television

☐0. Not at all ☐1. Several days ☐2. More than one-half of the days ☐3.Nearly every day

49. Moving or speaking so slowly that others could have noticed; or the opposite, being so fidgety or restless that you have been moving around a lot more than usual

☐0. Not at all ☐1. Several days ☐2. More than one-half of the days ☐3.Nearly every day

50. Thoughts that you would be better off dead, or of hurting yourself in some way

☐0. Not at all ☐1. Several days ☐2. More than one-half of the days ☐3.Nearly every day

51. PHQ-2 score

52. PHQ-9 score

**Safety**

53. Are there any weapons at home? ☐ Yes ☐ No

54. Do you think people of your age carry weapons to protect themselves? (knife, gun) ☐ Yes ☐ No

55. Do you get into physical fights in school or your neighborhood often?

☐No ☐Every day ☐once a week ☐once a month ☐once a year

56. Do you drive a motorcycle? ☐Yes ☐No

57. Do you use helmets while on a motorcycle? ☐Yes ☐No

58. Have you ever been touched in a way that you didn't want? ☐Yes ☐No

59. Have you skipped school in the last 12 months? ☐Yes ☐No

60. Have you been suspended or expelled from school? ☐Yes ☐No

61. Have you ever gotten into any physical fights at school? ☐Yes ☐No

62. Have you gotten into physical fights in your neighborhood, or other places? ☐Yes ☐No

63. Have you gotten in trouble with the police? ☐Yes ☐No

64. Have you been in situations where you destroyed property? ☐Yes ☐No

65. Have there been times when you stayed out very late without permission? ☐Yes ☐No

66. Have there been times when you have run away from home? ☐Yes ☐No

67. Conduct Disorder Concern ☐Yes ☐No
